# Mechanistic Targets and Phytochemical Strategies for Breaking the Obesity-Cancer Link

**DOI:** 10.3389/fonc.2013.00209

**Published:** 2013-08-19

**Authors:** Nikki A. Ford, Laura M. Lashinger, Emma H. Allott, Stephen D. Hursting

**Affiliations:** ^1^Department of Nutritional Sciences, University of Texas, Austin, TX, USA; ^2^Department of Urologic Surgery, Duke University Medical Center, Durham, NC, USA; ^3^Department of Molecular Carcinogenesis, University of Texas-MD Anderson Cancer Center, Smithville, TX, USA

**Keywords:** obesity, metabolism, cancer prevention, phytochemicals, inflammation, mTOR pathway

## Abstract

The prevalence of obesity, an established risk and progression factor for many cancers, has increased dramatically in many countries over the past three decades. Worldwide, an estimated 600 million adults are currently obese. Thus, a better understanding of the mechanistic links between obesity and cancer is urgently needed to identify intervention targets and strategies to offset the procancer effects of obesity. This review synthesizes the evidence on key biological mechanisms underlying the obesity-cancer association, with particular emphasis on obesity-associated enhancements in growth factor signaling, inflammation, and perturbations in the tumor microenvironment. These interrelated pathways and processes that are aberrantly regulated in obese individuals represent mechanism-based targets for disrupting the obesity-cancer link using phytochemicals.

## Introduction

An estimated 600 million adults throughout the world are obese, and the majority of these obese individuals meet the criteria for the metabolic syndrome, a state of metabolic dysregulation characterized by increased waist circumference, elevated fasting glucose levels, hypertension, and hypertriglyceridemia (1, 2). Increased circulating levels of insulin, bioavailable insulin-like growth factor (IGF)-1, leptin, inflammatory factors, and vascular integrity-related factors such as vascular endothelial growth factor (VEGF) and plasminogen activator inhibitor (PAI)-1, are typically observed in obese individuals (3–6). Through these, and likely other, mediators and their interacting pathways and processes, obesity increases the risk and/or worsens the outcome of several chronic diseases (3, 5) including cardiovascular disease, type II diabetes, and the focus of this review, cancer.

Obesity prevention or reversal is a major part of several evidence-based cancer prevention guidelines (7). Approximately 25% of cancer deaths in the US and United Kingdom are attributed to being overweight and obese (8), with the strongest evidence for endometrial, postmenopausal breast, colon, renal cell carcinoma, liver, gallbladder, esophageal adenocarcinoma, and pancreatic cancer, and mounting evidence for cervical, ovarian, prostate (prognosis, but not overall risk), and stomach cancer (7). This review focuses on possible mechanisms underlying the associations between obesity and cancer, with emphasis on obesity-associated enhancements in growth factor signaling, inflammatory processes, vascular perturbations, and microenvironmental disruptions, all linked with increased cancer susceptibility and poor prognosis. We will also discuss the potential for using phytochemicals known to modulate one or more of these energy balance-responsive pathways to contribute toward prevention or control of obesity-related cancers.

## Growth Signal Dysregulation

### Insulin and IGF-1, and their downstream signals

Hyperinsulinemia and/or hyperglycemia are hallmarks of the obese state and are associated with insulin resistance, aberrant glucose metabolism, chronic inflammation, and the production of other metabolic hormones such as IGF-1, leptin, and adiponectin (9). Insulin is a peptide hormone produced by the beta cells of the pancreas and released in response to increased blood glucose. IGF-1 is a peptide growth factor that shares ∼50% sequence homology with insulin and is produced primarily by the liver following stimulation by growth hormone, although hyperinsulinemia and hyperglycemia can lead to increased hepatic IGF-1 production independent of growth hormone signaling. Circulating IGF-1 is typically bound to IGF binding proteins (IGFBPs) that regulate IGF-1 bioavailability and modulate growth and survival signals either directly or by repressing the IGF-1R (10). With obesity, the amount of bioavailable IGF-1 increases, possibly via hyperglycemia-induced suppression of IGFBP synthesis and/or hyperinsulinemia-induced promotion of hepatic growth hormone receptor expression and IGF-1 synthesis (10, 11). Elevated circulating IGF-1 is an established risk factor for many obesity-associated cancer types (11).

In contrast, the enhanced insulin sensitivity and normalized glucose levels in response to a calorie restriction (CR) regimen, relative to a control or diet-induced obesity (DIO) regimen, results in lowered serum insulin and IGF-1, and increased IGFBP production, particularly IGFBP1 and 3 (and hence low levels of bioavailable IGF-1) (11). The CR-induced reduction in glucose may also have direct anticancer effects. In cancer cells, mitochondrial metabolism of glucose is reprogrammed to meet the demands of macromolecular synthesis required for cellular proliferation. This metabolic switch of glucose metabolism from oxidative phosphorylation to oxidative glycolysis (called the Warburg effect) is now understood to be necessary to supply sufficient nucleotides, lipids, and proteins for daughter cell production (10). Cancer cells do this, however, at the expense of substrate inflexibility relative to normal cells, as the increased proliferation rate associated with most cancer cells can only be sustained by a constant supply of the necessary building blocks derived from the flux of glucose carbons through glycolysis. Thus, it is possible that precancerous or cancer cells undergoing this metabolic reprogram, and hence developing a glucose addiction, may have heightened sensitivity to alterations in glucose levels, as occurs with obesity and CR.

The phospatidylinositol-3-kinase (PI3K)/Akt pathway, downstream of the insulin receptor and IGF-1R, comprises a signaling network that regulates and integrates cellular growth, survival, and metabolism. Cantley and colleagues (12) established that this signaling cascade is one of the most commonly altered pathways in human epithelial tumors. Engagement of the PI3K/Akt pathway results in production of both intracellular and extracellular cues concerning substrate availability, growth factor supply, and levels of other factors that impact cell survival, growth, proliferation, and metabolism. Activation of receptor tyrosine kinases, such as the insulin receptor or IGF-1R, stimulates PI3K to produce lipid messengers that facilitate activation of the Akt cascade. Akt regulates the mammalian target of rapamycin (mTOR) (13), thus regulating cell growth, proliferation, and survival through downstream mediators. mTOR activation is inhibited by increased AMP-activated kinase (AMPK) under low nutrient conditions (14, 15). Increased activation of mTOR is common in tumors and many normal tissues from obese and/or diabetic mice, and specific mTOR inhibitors block the tumor-enhancing effects of obesity in mouse models (15–17).

### Leptin, adiponectin, and their ratio

Leptin, a peptide hormone produced predominantly by adipocytes, functions as an energy sensor to signal to the hypothalamus to reduce appetite. Insulin, glucocorticoids, tumor necrosis factor-alpha (TNF-α), and estrogens all stimulate leptin release (18). In the obese state, adipose tissue overproduces leptin, and the brain eventually becomes resistant to this satiety signal. In addition to its role in regulating appetite, leptin has direct pro-tumorigenic effects on peripheral tissues, as well as a role in modulating immune function, cytokine production, angiogenesis, carcinogenesis, and other biological processes (18). The leptin receptor has similar homology to class I cytokines that signal through the janus kinase and signal transducer activator of transcription (JAK/STAT) pathway that is often dysregulated in cancer (19).

Although adiponectin is a hormone mainly secreted from visceral adipose tissue, its serum levels, in contrast with serum leptin, negatively correlate with adiposity. Adiponectin functions to counter the metabolic program associated with obesity and hyperleptinemia by modulating glucose metabolism, increasing fatty acid oxidation and insulin sensitivity, and decreasing production of inflammatory cytokines (20). The possible mechanisms through which adiponectin exerts anticancer effects may include increasing insulin sensitivity, and decreasing insulin/IGF-1 and mTOR signaling via activation of AMPK. Adiponectin also reduces pro-inflammatory cytokine expression via inhibition of the nuclear factor kappa-light-chain-enhancer of activated B-cells (NF-κB) (20–22).

*In vitro*, animal and epidemiologic evidence linking leptin (21, 23–26) or adiponectin (21, 27–31) individually to cancer risk is mixed. Associations between the adiponectin-to-leptin ratio and the metabolic syndrome and several cancers (32–34) have also been reported, but there is insufficient data thus far to assess the strength of this relationship.

## Chronic Inflammation

### Cytokines and crosstalk between epithelial cells, adipocytes, and macrophages

Obesity and metabolic syndrome are associated with a low-grade, chronic state of inflammation characterized by increased circulating free fatty acids and chemoattraction of immune cells (such as macrophages that also produce inflammatory mediators) into the local milieu of expanded adipose tissue (35–37). These effects are further amplified by the release of inflammatory cytokines such as interleukin (IL)-1β, IL-6, TNF-α, and monocyte chemoattractant protein (MCP)-1. Hypertrophic adipocytes can enlarge past the point of effective oxygen diffusion, which results in hypoxia and eventually necrosis leading to further infiltration of scavenging macrophages and formation of crown-like structures. Free fatty acids escape the engorged/necrotic adipocytes and deposit in other tissues, which in turn promotes insulin resistance and diabetes (through downregulation of insulin receptors and glucose transporters), hypertension, and fatty liver disease and also activates signaling molecules involved in epithelial carcinogenesis, such as NF-κB (35–37).

NF-κB is a transcription factor that is activated in response to bacterial and viral stimuli, growth factors, and inflammatory molecules (e.g., TNF-α, IL-6, and IL-1β), and is responsible for inducing gene expression associated with cell proliferation, apoptosis, inflammation, metastasis, and angiogenesis (35–37). Activation of NF-κB is a common characteristic of many tumors and is associated with insulin resistance and elevated circulating levels of leptin, insulin, and/or IGF-1 (37–40).

### Inflammation and cancer

The link between chronic inflammation and cancer development was first reported more than 100 years ago by Rudolph Virchow, who observed an abundance of leukocytes in neoplastic tissue (41). Now, several tissue-specific inflammatory lesions are established neoplastic precursors for invasive cancer, including gastritis for gastric cancer, inflammatory bowel disease for colon cancer, and pancreatitis for pancreatic cancer (42, 43).

Tumor microenvironments are composed of multiple cell types including epithelial cells, fibroblasts, mast cells, and cells of the innate and adaptive immune system (43, 44). As discussed previously, macrophages, which are classically activated in the obese state, infiltrate tumors and amplify the inflammatory tumor microenvironment through production of pro-inflammatory cytokines, prostaglandins, and angiogenic factors (37, 44). Another important cancer-related inflammatory mediator is cyclooxygenase (COX)-2, an enzyme that is upregulated in most tumors and catalyzes the synthesis of the potent inflammatory lipid metabolite, prostaglandin E_2_. COX-2 overexpression is an indicator of poor prognosis in multiple cancer types (45).

## Vascular Integrity-Related Factors

### Plasminogen activator inhibitor-1

Plasminogen activator inhibitor-1 is a serine protease inhibitor produced by endothelial cells, stromal cells, and adipocytes in visceral white adipose tissue (46). Increased circulating PAI-1 levels, frequently found in obese subjects, are associated with increased risk of atherogenesis and cardiovascular disease, diabetes, and several cancers (4, 46). PAI-1, through its inhibition of urokinase-type and tissue-type plasminogen activators, regulates fibrinolysis and integrity of the extracellular matrix (ECM). Furthermore, PAI-1 can modulate cell adhesion through decreasing cell binding to the ECM protein, vitronectin, thus promoting tumor cell detachment from the ECM (46). PAI-1 is also involved in angiogenesis and thus may contribute to obesity-driven tumor cell growth, invasion, and metastasis (4).

### Vascular endothelial growth factor

Vascular endothelial growth factor, a heparin-binding glycoprotein produced by adipocytes and tumor cells, has angiogenic, mitogenic, and vascular permeability-enhancing activities specific for endothelial cells (47). Circulating levels of VEGF are increased in obese, relative to lean humans and animals, and increased tumoral expression of VEGF is associated with poor prognosis in several obesity-related cancers (48). The need for nutrients and oxygen triggers tumor cells to produce VEGF, which leads to the formation of new blood vessels to nourish the rapidly growing tumor and facilitate the metastatic spread of tumor cells (49).

Adipocytes communicate with endothelial cells by producing a variety of proangiogenic and vascular permeability-enhancing factors. These include VEGF, IGF-1, PAI-1, leptin, hepatocyte growth factor, and fibroblast growth factor-2 (49). In the obese, non-tumor setting, these factors stimulate neovascularization in support of the expanding fat mass. These adipose-derived factors may also contribute to obesity-associated enhancement of tumor angiogenesis. However, the relative contributions of tumor-derived, versus adipocyte-derived, proangiogenic factors in tumor development, progression, and metastasis remain unclear.

## Phytochemical Modulation of Obesity-Cancer Links

Since the biological effects of a broad spectrum of phytochemicals have been reviewed in other articles in this special issue, we will focus here on some specific examples of phytochemicals that target the key pathways underlying obesity-cancer associations.

### Resveratrol

*Resveratrol*, chemically known as 3,5,4′-trihydroxystilbene, is a naturally occurring polyphenolic compound present in grapes, berries, peanuts, and red wine. It is believed to be responsible for the so called “French paradox,” in which the consumption of red wine has been shown to reduce the mortality rates from cardiovascular disease and certain cancers. Resveratrol is an established anti-inflammatory agent that inhibits initiation and growth of numerous cancer types including breast, prostate, colon, and liver (50). Recent clinical studies demonstrate resveratrol improves glycemic control in diabetic patients (51) and reduces inflammatory signaling through TNF-α, IL-6, C-reactive protein, and NFκB pathways (52). Furthermore, at physiologic concentrations resveratrol (possibly by activating the sirtuin pathway) counteracts activation of tumoral COX-2 and NFκB (53). Recently published reports suggest that resveratrol mimics some of the effects of CR on lifespan in worms and other model organisms, apparently by inhibiting inflammation and the Akt/mTOR pathway (54, 55).

### Ursolic acid

*Ursolic acid* is a triterpenoid found in fairly high levels in rosemary and in smaller amounts in apples and other fruits and vegetables. Ursolic acid has been shown to inhibit carcinogen and tumor promoter-induced inflammation, hyperplasia, and tumor formation in multiple models (56–59). Ursolic acid is a very potent anti-inflammatory and insulin sensitizing agent with reported activities against the effects of obesity, such as the ability to counteract NF-κB, COX-2, and Akt activity (53, 59). Furthermore, we reported that a diet containing 0.1% (w/w) ursolic acid suppressed MMTV-Wnt-1 murine mammary tumor growth, decreased insulin and IGF-1, and decreased activation of the mTOR pathway (59). Thus, like resveratrol, ursolic acid is thought to mimic some of the effects of CR. Ursolic acid was also shown to inhibit tumor-associated capillary formation in mice through inhibition of VEGF and other inflammatory growth factors (60).

### Curcumin

*Curcumin*, also known as diferuloylmethane, is an anti-inflammatory agent that gives yellow color to turmeric used in curry powder. Curcumin has been shown to have chemopreventative properties across several cancer types (61). These anticancer effects are modulated through suppression of several inflammatory and growth signaling pathways including NFκB, Stat3, COX-2, Akt, and mTOR (53, 62). Furthermore, inhibition of COX-2 by curcumin also results in suppression of VEGF-mediated angiogenesis (63). By targeting these pathways, curcumin has been shown to reverse hyperglycemia, hyperlipidemia, and other symptoms related to obesity and therefore reduce the growth and development of obesity-associated cancers (64, 65).

### Quercetin

*Quercetin* is found commonly in citrus fruits, onions, tea, and red wine and possesses significant antioxidant activity, like other polyphenols (curcumin and resveratrol). Quercetin targets many of the key pathways involved in tumor initiation, development, growth, and metastases. Specifically, it can modulate the Akt/mTOR pathway, COX-2 expression, NFκB signaling, TNFα expression, and VEGF expression (53, 66–68). A phase I clinical study in cancer patients showed that quercetin effectively inhibits tyrosine kinases involved in growth, inflammation, and cell signaling (69).

## Conclusion

**Figure 1:**
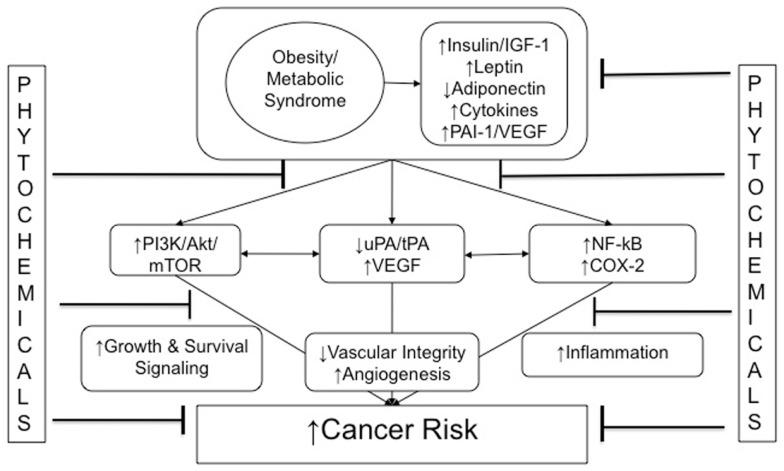
**Obesity and cancer: mechanistic targets for phytochemical interventions**. An arrow preceding text denotes a directional effect (e.g., activity or concentration). Abbreviations: IGF-1, insulin-like growth factor-1; ApN, adiponectin; PAI-1, plasminogen activator inhibitor-1; tPA, tissue-type plasminogen activator; uPA, urokinase-type plasminogen activator; VEGF, vascular endothelial growth factor; PI3K, phosphoinositide-3-kinase; NF-κB, nuclear factor κB; COX-2, cyclooxygenase-2; EMT, epithelial-to-mesenchymal transition.

As summarized in Figure 1, multiple hormones, growth factors, cytokines, and other mediators associated with the metabolic perturbations of the obese state enable crosstalk between macrophages, adipocytes, endothelial cells, and epithelial cells. These obesity-associated factors contribute to cancer-related processes (including growth signaling, inflammation, and vascular alterations). Components of these interrelated processes and pathways represent promising mechanism-based targets for phytochemical interventions, with the goal of breaking the links between obesity (and its metabolic dysregulation) and cancer.

## Conflict of Interest Statement

The authors declare that the research was conducted in the absence of any commercial or financial relationships that could be construed as a potential conflict of interest.
